# Supplementary material for: Deletion of wfs1 Impairs Oligodendrocyte Precursor Cells Dorsal Distribution and Myelination Through the wfs1-hmgcs1 Axis in Zebrafish
Source: Biology (Basel). 2026 Aug 16;15(16):1402. doi: 10.3390/biology15161402 (PMC13510151; doi:10.3390/biology15161402)
Supplement: Supplementary file 1 [file biology-15-01402-s001.zip › File S1-Transcriptome sequencing data and differentially expressed genes (DEGs) in wfs1-- zebrafish at 5 dpf/2.KEGG/wfs1KOvsWT/GSEA/WT/gsea_report_for_WT_1700447271053.html]

Report for WT 1700447271053 [GSEA]


| GS   follow link to MSigDB | GS DETAILS | SIZE | ES | NES | NOM p-val | FDR q-val | FWER p-val | RANK AT MAX | LEADING EDGE || 1 | OTHER\_GLYCAN\_DEGRADATION(DRE00511) | Details ... | 28 | -0.55 | -1.53 | 0.032 | 1.000 | 0.557 | 6371 | tags=43%, list=20%, signal=54% |
| 2 | ENDOCYTOSIS(DRE04144) | Details ... | 417 | -0.25 | -1.49 | 0.000 | 0.869 | 0.722 | 3921 | tags=12%, list=12%, signal=13% |
| 3 | SELENOCOMPOUND\_METABOLISM(DRE00450) | Details ... | 22 | -0.40 | -1.49 | 0.034 | 0.601 | 0.722 | 1854 | tags=23%, list=6%, signal=24% |
| 4 | FERROPTOSIS(DRE04216) | Details ... | 73 | -0.32 | -1.46 | 0.033 | 0.572 | 0.810 | 5376 | tags=25%, list=17%, signal=30% |
| 5 | NECROPTOSIS(DRE04217) | Details ... | 215 | -0.29 | -1.43 | 0.000 | 0.534 | 0.853 | 3170 | tags=13%, list=10%, signal=14% |
| 6 | TIGHT\_JUNCTION(DRE04530) | Details ... | 300 | -0.23 | -1.34 | 0.000 | 0.795 | 0.926 | 3869 | tags=12%, list=12%, signal=14% |
| 7 | FRUCTOSE\_AND\_MANNOSE\_METABOLISM(DRE00051) | Details ... | 54 | -0.35 | -1.33 | 0.026 | 0.701 | 0.940 | 4374 | tags=28%, list=14%, signal=32% |
| 8 | UBIQUITIN\_MEDIATED\_PROTEOLYSIS(DRE04120) | Details ... | 199 | -0.24 | -1.32 | 0.028 | 0.644 | 0.940 | 3501 | tags=11%, list=11%, signal=12% |
| 9 | APOPTOSIS(DRE04210) | Details ... | 232 | -0.24 | -1.32 | 0.026 | 0.586 | 0.956 | 4772 | tags=19%, list=15%, signal=23% |
| 10 | MANNOSE\_TYPE\_O\_GLYCAN\_BIOSYNTHESIS(DRE00515) | Details ... | 39 | -0.35 | -1.31 | 0.133 | 0.549 | 0.956 | 5556 | tags=21%, list=17%, signal=25% |
| 11 | PROTEIN\_EXPORT(DRE03060) | Details ... | 25 | -0.32 | -1.29 | 0.178 | 0.586 | 0.987 | 861 | tags=8%, list=3%, signal=8% |
| 12 | MTOR\_SIGNALING\_PATHWAY(DRE04150) | Details ... | 251 | -0.27 | -1.28 | 0.069 | 0.552 | 0.987 | 6233 | tags=24%, list=20%, signal=29% |
| 13 | BASE\_EXCISION\_REPAIR(DRE03410) | Details ... | 58 | -0.31 | -1.28 | 0.144 | 0.515 | 0.987 | 3099 | tags=12%, list=10%, signal=13% |
| 14 | BIOSYNTHESIS\_OF\_AMINO\_ACIDS(DRE01230) | Details ... | 109 | -0.28 | -1.27 | 0.126 | 0.496 | 0.987 | 4385 | tags=23%, list=14%, signal=27% |
| 15 | AUTOPHAGY\_\_\_OTHER(DRE04136) | Details ... | 41 | -0.30 | -1.25 | 0.195 | 0.519 | 0.987 | 448 | tags=5%, list=1%, signal=5% |
| 16 | REGULATION\_OF\_ACTIN\_CYTOSKELETON(DRE04810) | Details ... | 404 | -0.23 | -1.24 | 0.044 | 0.501 | 0.987 | 3920 | tags=15%, list=12%, signal=16% |
| 17 | LYSINE\_DEGRADATION(DRE00310) | Details ... | 102 | -0.24 | -1.23 | 0.109 | 0.519 | 1.000 | 3248 | tags=11%, list=10%, signal=12% |
| 18 | MITOPHAGY\_\_\_ANIMAL(DRE04137) | Details ... | 116 | -0.26 | -1.23 | 0.132 | 0.496 | 1.000 | 4338 | tags=19%, list=14%, signal=22% |
| 19 | NOD\_LIKE\_RECEPTOR\_SIGNALING\_PATHWAY(DRE04621) | Details ... | 205 | -0.24 | -1.22 | 0.020 | 0.473 | 1.000 | 3135 | tags=10%, list=10%, signal=11% |
| 20 | SPHINGOLIPID\_METABOLISM(DRE00600) | Details ... | 89 | -0.26 | -1.22 | 0.098 | 0.466 | 1.000 | 2443 | tags=8%, list=8%, signal=8% |
| 21 | BETA\_ALANINE\_METABOLISM(DRE00410) | Details ... | 41 | -0.29 | -1.21 | 0.239 | 0.472 | 1.000 | 3868 | tags=20%, list=12%, signal=22% |
| 22 | 2\_OXOCARBOXYLIC\_ACID\_METABOLISM(DRE01210) | Details ... | 30 | -0.35 | -1.16 | 0.235 | 0.554 | 1.000 | 1258 | tags=10%, list=4%, signal=10% |
| 23 | DNA\_REPLICATION(DRE03030) | Details ... | 48 | -0.31 | -1.16 | 0.296 | 0.543 | 1.000 | 1499 | tags=8%, list=5%, signal=9% |
| 24 | VIRAL\_LIFE\_CYCLE\_\_\_HIV\_1(DRE03250) | Details ... | 83 | -0.26 | -1.16 | 0.227 | 0.522 | 1.000 | 3272 | tags=13%, list=10%, signal=15% |
| 25 | VEGF\_SIGNALING\_PATHWAY(DRE04370) | Details ... | 104 | -0.26 | -1.14 | 0.210 | 0.534 | 1.000 | 5420 | tags=24%, list=17%, signal=29% |
| 26 | BASAL\_TRANSCRIPTION\_FACTORS(DRE03022) | Details ... | 45 | -0.25 | -1.13 | 0.204 | 0.549 | 1.000 | 5926 | tags=27%, list=19%, signal=33% |
| 27 | HERPES\_SIMPLEX\_VIRUS\_1\_INFECTION(DRE05168) | Details ... | 220 | -0.21 | -1.13 | 0.137 | 0.531 | 1.000 | 3729 | tags=12%, list=12%, signal=14% |
| 28 | CALCIUM\_SIGNALING\_PATHWAY(DRE04020) | Details ... | 450 | -0.23 | -1.12 | 0.185 | 0.516 | 1.000 | 5328 | tags=20%, list=17%, signal=23% |
| 29 | GAP\_JUNCTION(DRE04540) | Details ... | 160 | -0.24 | -1.11 | 0.252 | 0.531 | 1.000 | 4038 | tags=14%, list=13%, signal=16% |
| 30 | AMINOACYL\_TRNA\_BIOSYNTHESIS(DRE00970) | Details ... | 51 | -0.28 | -1.11 | 0.358 | 0.520 | 1.000 | 10351 | tags=53%, list=32%, signal=78% |
| 31 | MOTOR\_PROTEINS(DRE04814) | Details ... | 305 | -0.21 | -1.10 | 0.373 | 0.519 | 1.000 | 6492 | tags=23%, list=20%, signal=29% |
| 32 | GLYCOLYSIS\_\_\_GLUCONEOGENESIS(DRE00010) | Details ... | 103 | -0.28 | -1.08 | 0.292 | 0.544 | 1.000 | 4213 | tags=22%, list=13%, signal=26% |
| 33 | GLYCOSPHINGOLIPID\_BIOSYNTHESIS\_\_\_GANGLIO\_SERIES(DRE00604) | Details ... | 19 | -0.39 | -1.06 | 0.451 | 0.569 | 1.000 | 45 | tags=5%, list=0%, signal=5% |
| 34 | MISMATCH\_REPAIR(DRE03430) | Details ... | 30 | -0.31 | -1.05 | 0.435 | 0.579 | 1.000 | 1052 | tags=7%, list=3%, signal=7% |
| 35 | CARDIAC\_MUSCLE\_CONTRACTION(DRE04260) | Details ... | 173 | -0.25 | -1.04 | 0.427 | 0.576 | 1.000 | 6492 | tags=27%, list=20%, signal=33% |
| 36 | TOLL\_LIKE\_RECEPTOR\_SIGNALING\_PATHWAY(DRE04620) | Details ... | 121 | -0.22 | -1.04 | 0.360 | 0.571 | 1.000 | 3746 | tags=15%, list=12%, signal=17% |
| 37 | ALANINE\_\_ASPARTATE\_AND\_GLUTAMATE\_METABOLISM(DRE00250) | Details ... | 57 | -0.26 | -1.03 | 0.330 | 0.571 | 1.000 | 4130 | tags=16%, list=13%, signal=18% |
| 38 | HISTIDINE\_METABOLISM(DRE00340) | Details ... | 30 | -0.28 | -1.03 | 0.382 | 0.569 | 1.000 | 6169 | tags=33%, list=19%, signal=41% |
| 39 | WNT\_SIGNALING\_PATHWAY(DRE04310) | Details ... | 278 | -0.21 | -1.02 | 0.318 | 0.566 | 1.000 | 3214 | tags=10%, list=10%, signal=11% |
| 40 | ERBB\_SIGNALING\_PATHWAY(DRE04012) | Details ... | 127 | -0.22 | -0.98 | 0.493 | 0.636 | 1.000 | 5420 | tags=18%, list=17%, signal=22% |
| 41 | RNA\_POLYMERASE(DRE03020) | Details ... | 42 | -0.30 | -0.95 | 0.479 | 0.673 | 1.000 | 5224 | tags=17%, list=16%, signal=20% |
| 42 | GLYCOSPHINGOLIPID\_BIOSYNTHESIS\_\_\_LACTO\_AND\_NEOLACTO\_SERIES(DRE00601) | Details ... | 42 | -0.22 | -0.90 | 0.575 | 0.776 | 1.000 | 3610 | tags=12%, list=11%, signal=13% |
| 43 | NEUROACTIVE\_LIGAND\_RECEPTOR\_INTERACTION(DRE04080) | Details ... | 637 | -0.21 | -0.88 | 0.732 | 0.806 | 1.000 | 5808 | tags=21%, list=18%, signal=25% |
| 44 | NUCLEOTIDE\_EXCISION\_REPAIR(DRE03420) | Details ... | 68 | -0.21 | -0.88 | 0.639 | 0.788 | 1.000 | 7408 | tags=24%, list=23%, signal=31% |
| 45 | PRIMARY\_BILE\_ACID\_BIOSYNTHESIS(DRE00120) | Details ... | 31 | -0.31 | -0.87 | 0.569 | 0.782 | 1.000 | 1806 | tags=13%, list=6%, signal=14% |
| 46 | OXIDATIVE\_PHOSPHORYLATION(DRE00190) | Details ... | 171 | -0.21 | -0.86 | 0.544 | 0.793 | 1.000 | 9801 | tags=44%, list=31%, signal=63% |
| 47 | GLYCOSYLPHOSPHATIDYLINOSITOL\_(GPI)\_ANCHOR\_BIOSYNTHESIS(DRE00563) | Details ... | 25 | -0.27 | -0.85 | 0.751 | 0.790 | 1.000 | 3226 | tags=12%, list=10%, signal=13% |
| 48 | RIBOSOME(DRE03010) | Details ... | 161 | -0.22 | -0.83 | 0.546 | 0.820 | 1.000 | 10568 | tags=41%, list=33%, signal=61% |
| 49 | ASCORBATE\_AND\_ALDARATE\_METABOLISM(DRE00053) | Details ... | 66 | -0.28 | -0.80 | 0.603 | 0.864 | 1.000 | 2070 | tags=15%, list=6%, signal=16% |
| 50 | ARGININE\_BIOSYNTHESIS(DRE00220) | Details ... | 35 | -0.24 | -0.78 | 0.701 | 0.868 | 1.000 | 6886 | tags=29%, list=22%, signal=36% |
| 51 | GLYCOSAMINOGLYCAN\_DEGRADATION(DRE00531) |  | 27 | -0.20 | -0.72 | 0.820 | 0.923 | 1.000 | 6008 | tags=26%, list=19%, signal=32% |
| 52 | N\_GLYCAN\_BIOSYNTHESIS(DRE00510) |  | 68 | -0.16 | -0.71 | 0.865 | 0.917 | 1.000 | 6199 | tags=15%, list=19%, signal=18% |
| 53 | HEDGEHOG\_SIGNALING\_PATHWAY(DRE04340) |  | 79 | -0.14 | -0.65 | 0.965 | 0.945 | 1.000 | 2774 | tags=6%, list=9%, signal=7% |
Table: Gene sets enriched in phenotype
 **WT (4 samples)
 **[plain text format]****
